# Protura are unique: first evidence of specialized feeding on ectomycorrhizal fungi in soil invertebrates

**DOI:** 10.1186/s12898-019-0227-y

**Published:** 2019-02-22

**Authors:** Sarah L. Bluhm, Anton M. Potapov, Julia Shrubovych, Silke Ammerschubert, Andrea Polle, Stefan Scheu

**Affiliations:** 1 J.F. Blumenbach Institute of Zoology and Anthropology, Animal Ecology, University of Göttingen, Untere Karspüle 2, 37073 Göttingen, Germany; 2 Russian Academy of Sciences, A.N. Severtsov Institute of Ecology and Evolution, Leninsky Prospect 33, Moscow, 119071 Russia; 3 Institute of Systematics and Evolution of Animals, Polish Academy of Sciences, ul. Slawkowska 17, 31-016 Krakow, Poland; 4 State Museum of Natural History, Ukrainian Academy of Sciences, Teatral’na St. 18, L’viv, UA 79008 Ukraine; 5 Institute of Soil Biology, Biology Centre of Czech Academy of Sciences, Na Sádkách 7, 370 05 České Budějovice, Czech Republic; 6 Büsgen Institute, Forest Botany and Tree Physiology, University of Göttingen, Büsgenweg 2, 37077 Göttingen, Germany; 7 Centre of Biodiversity and Sustainable Land Use, University of Göttingen, Von-Siebold-Str. 8, 37075 Göttingen, Germany

**Keywords:** *Acerentomon*, Carbon, Nitrogen, Nutrition biology, Mycorrhiza, Pulse labelling, Stable isotopes, Rhizosphere, Temperate forests, Carbon sequestration

## Abstract

**Background:**

Ectomycorrhizal fungi (ECM) play a central role in nutrient cycling in boreal and temperate forests, but their role in the soil food web remains little understood. One of the groups assumed to live as specialised mycorrhizal feeders are Protura, but experimental and field evidence is lacking. We used a combination of three methods to test if Protura are specialized mycorrhizal feeders and compared their trophic niche with other soil invertebrates. Using pulse labelling of young beech and ash seedlings we analysed the incorporation of ^13^C and ^15^N into *Acerentomon gallicum.* In addition, individuals of Protura from temperate forests were collected for the analysis of neutral lipid fatty acids and natural variations in stable isotope ratios.

**Results:**

Pulse labelling showed rapid incorporation of root-derived ^13^C, but no incorporation of root-derived ^15^N into *A. gallicum*. The transfer of ^13^C from lateral roots to ectomycorrhizal root tips was high, while it was low for ^15^N. Neutral lipid fatty acid (NLFA) analysis showed high amounts of bacterial marker (16:1ω7) and plant marker (16:0 and 18:1ω9) fatty acids but not of the fungal membrane lipid 18:2ω6,9 in *A. gallicum*. Natural variations in stable isotope ratios in Protura from a number of temperate forests were distinct from those of the great majority of other soil invertebrates, but remarkably similar to those of sporocarps of ECM fungi.

**Conclusions:**

Using three in situ methods, stable isotope labelling, neutral lipid fatty acid analysis and natural variations of stable isotope ratios, we showed that Protura predominantly feed on mycorrhizal hyphae via sucking up hyphal cytoplasm. Predominant feeding on ectomycorrhizal mycelia by Protura is an exception; the limited consumption of ECM by other soil invertebrates may contribute to carbon sequestration in temperate and boreal forests.

**Electronic supplementary material:**

The online version of this article (10.1186/s12898-019-0227-y) contains supplementary material, which is available to authorized users.

## Background

Ectomycorrhizal fungi (ECM) account for a large fraction of microbial biomass in forest soils [1–3]. They play a central role in element cycling, providing plants with nitrogen (N) and channelling large amounts of plant-derived carbon (C) into the soil [1, 4, 5] thereby affecting storage of C in soils [6]. By using in-growth bags, the production rate of the extramatrical mycelium of ECM in the upper 10 cm of forest soil was estimated to be about 160 kg dry matter ha^−1^ y^−1^ [1]. The high production suggests that mycorrhizal mycelium serve as an important food resource for soil invertebrates [7, 8], but recent evidence showed that consumption of ECM by soil invertebrates is limited [9].

Protura are tiny soil living hexapods that may reach densities of up to 90,000 individuals m^−2^ in temperate forests [10], but typically their density is lower with 200–7000 individuals m^−2^ not reaching densities of springtails and mites [11, 12]. They have been observed to feed on mycorrhizal hyphae via sucking up hyphal cytoplasm [13], but little is known about their nutritional biology [14] and experimental investigations on their food resources in situ are missing.

Trophic links in soil communities are increasingly studied by using isotope labelling, analysis of natural variations in stable isotope ratios and lipid analysis [15]. ^13^C and ^15^N isotopes can be used for pulse labelling of plant shoots and for tracking the incorporation of these elements into the belowground system [16]. Due to the close link of mycorrhizal fungi to recent photosynthates, stable isotope labelling can be used to investigate the role of ECM as a food resource for invertebrate consumers [8]. In turn, the natural abundance of ^13^C and ^15^N varies between saprotrophic fungi and ECM, a phenomenon termed ‘ECM—sap divide’ [17] and this allows distinguishing between animals, feeding on these two functional groups of fungi [9]. ECM are depleted in ^13^C and enriched in ^15^N as compared to saprotrophic fungi due to the different use of C and N sources as well as discrimination during the transfer of N from ECM to plant roots [18–20]. Further, fatty acid analysis allows to distinguish between plant, fungi and bacteria in the diet of soil invertebrates, since biomarker membrane lipids in the diet are incorporated into neutral fatty acids of consumers without major change (‘dietary rooting’; [21]).

In this study we used a combination of techniques to get insight into the nutritional biology of Protura in situ: first, we used pulse labelling of tree seedlings to investigate the incorporation of root-derived C and N into *Acerentomon gallicum* Ionesco, 1933, a common Protura species in forest ecosystems; second, we analysed the neutral lipid fatty acid (NLFA) composition of *A. gallicum* to uncover its association to the bacterial, fungal or plant based soil energy channel; third, we measured natural variations in stable isotope ratios in a number of species of Protura in a range of temperate forests to relate their trophic niche to ECM and other soil invertebrates. Following the assumption that Protura specialised in feeding on mycorrhizal mycelia, we hypothesised that (i) Protura will incorporate recently assimilated plant C that is transferred to ECM, but not plant N, that is taken up by ECM from the soil, (ii) NLFA of Protura will comprise predominantly fungal biomarkers reflecting consumption of ECM, and (iii) natural abundances of ^13^C and ^15^N in Protura will resemble those of mycorrhizal fungi, but will be distinct from other soil invertebrates.

## Results

In the rhizosphere of labelled beech, *A. gallicum* had significantly higher δ^13^C signatures as compared to the control (4597 ± 2655 and − 26.8 ± 0.4‰, respectively; F_1,9_ = 9.73, p = 0.012), but δ^15^N signatures did not differ significantly from the control (respective values of 4.0 ± 2.2 and 3.7 ± 1.0‰; F_1,9_ = 0.41, p = 0.54). ECM root tips were significantly more enriched in ^13^C (3771 ± 4514 Δε‰) than lateral roots (2428 ± 2380 Δε‰, F_1,32_ = 6.52, p = 0.016), but ECM root tips were less enriched in ^15^N (51.3 ± 76.7 Δε‰) as compared to lateral roots (134.4 ± 144.5 Δε‰, F_1,32_ = 46.21, p < 0.001). Around 30% of the beech root tips were colonized by species of the family Pezizaceae (*Pachyphlodes conglomerata* (Berk. & Broome) Doweld, 2013) and up to 20% by uncultured EM, order Heliotales. Other ECM species such *Tomentella punicea* (Alb. & Schwein.) J. Schröt., 1888, *Cenococcum geophilum* Fries, 1829 were also found but at lower frequencies.

**Fig. 1:**
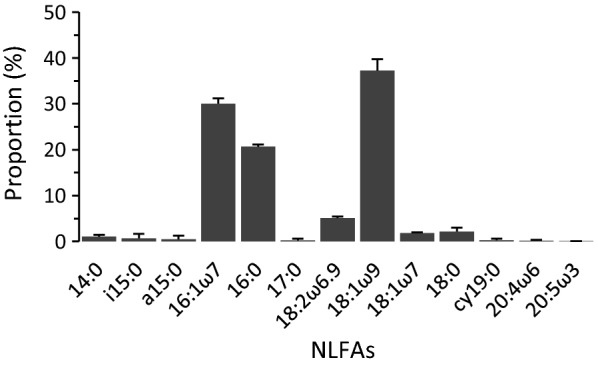
Composition of neutral lipid fatty acids (NLFAs) in *Acerentomon gallicum* as percentages of total NLFAs including bacterial (16:1ω7, 18:1ω7, cy19:0), fungal (18:2ω6,9) and plant biomarker fatty acids (18:1ω9) [21] (means and standard deviations)

Statistical analysis was not carried out due to low sample numbers in ash, but in labelled treatments ^13^C signatures of *A. gallicum* in the beech rhizosphere (4624 ± 2655 Δε‰) markedly exceeded that in the ash rhizosphere (23.7 ± 3.6 Δε‰), whereas ^15^N enrichment was low in both tree species with an overall mean of 0.7 ± 1.1 Δε‰. The most abundant NLFA in *A. gallicum* was 18:1ω9 comprising 37.3 ± 2.4% of total NLFAs followed by 16:1ω7 (30.0 ± 1.2%) and 16:0 (20.7 ± 0.5%), while 18:2ω6,9 only made up 5.1 ± 0.4% (Fig. 1).

Litter-normalized Δ^15^N values were similar in *A. gallicum* and *Eosentomon* spp. but the variation was larger in the latter (6.2 ± 0.9 and 6.7 ± 3.0‰, respectively). Litter-normalized Δ^13^C values were higher in *A. gallicum* than in *Eosentomon* spp. (3.0 ± 0.1 and 2.1 ± 0.7‰, respectively; *t*-test p < 0.001). Litter-normalized Δ^13^C and Δ^15^N values in the single sample of *Acerentulus* sp. were 3.4 and 11.6‰, respectively.

**Fig. 2:**
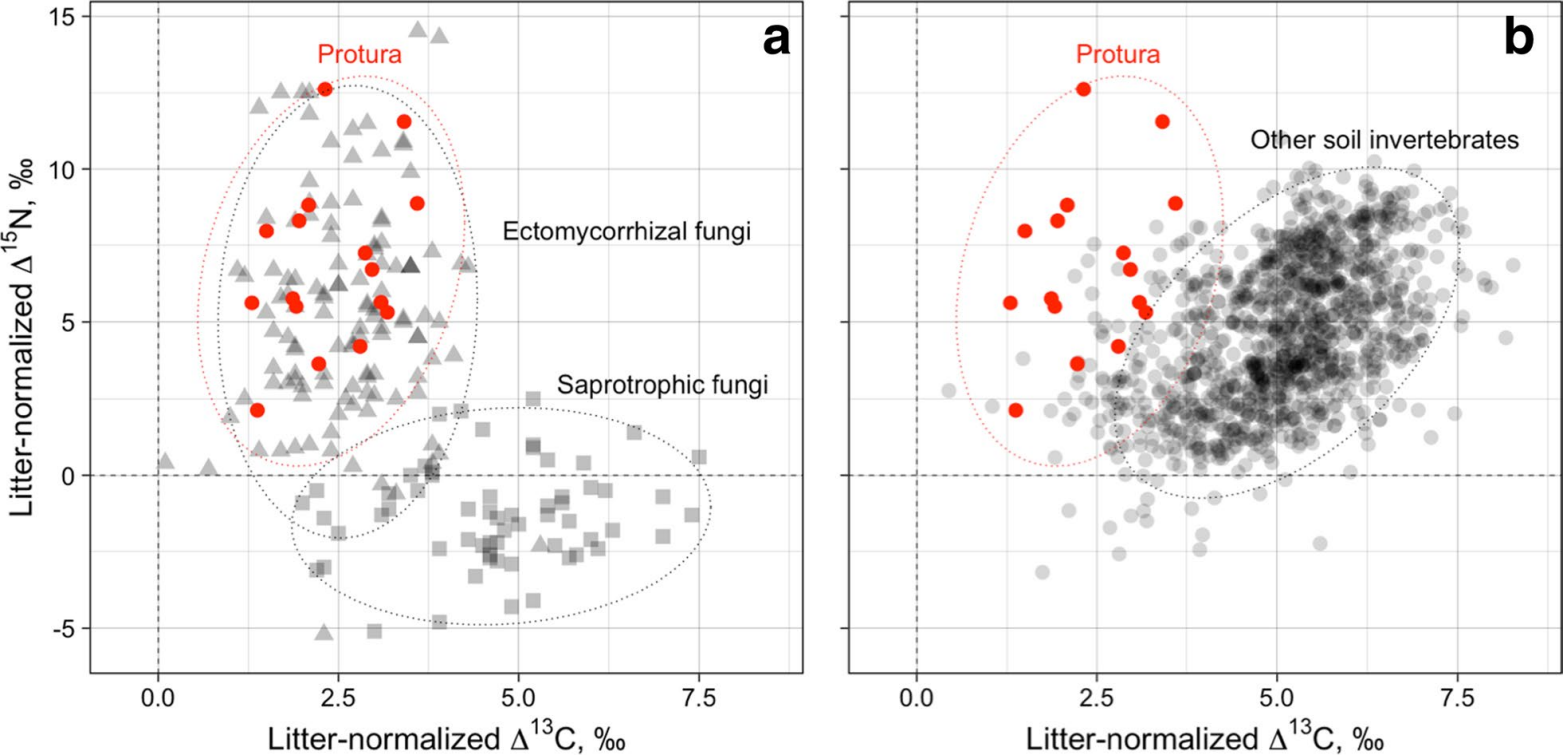
Natural variations in stable isotope ratios (litter-normalized Δ^13^C and Δ^15^N values) of Protura as compared to other invertebrates. Each point represents mean stable isotope ratios of individual species in local communities; ellipses represent 95% confidence ranges. Note: the stable isotope niche of Protura resembles that of mycorrhizal fungi and differs from that of saprotrophic fungi (**a**) as well as the majority of other soil invertebrates (**b**). Data on saprotrophic fungi are based on fungal sporocarps from coniferous forests [56], data on invertebrates are based on soil animals from beech and coniferous forests [46]

Litter-normalized Δ^15^N and Δ^13^C values of Protura were very similar to those of sporocarps of mycorrhizal, but not to sporocarps of saprotrophic fungi (Fig. 2a). Further, the isotopic niche of Protura overlapped little with that of virtually all other groups of soil invertebrates (Fig. 2b). Among decomposers, litter-normalized Δ^15^N and Δ^13^C values were similar to few samples of *Damaeus riparius* Nicolet, 1855 (Oribatida, average values of Δ^15^N and Δ^13^C of 3.1 ± 1.1‰ and 3.3 ± 0.7‰, respectively) and *Athous* spp. (Elateridae; average Δ^15^N and Δ^13^C values of 6.6 ± 1.1‰ and 5.5 ± 1.4‰, respectively). Further, litter-normalized Δ^15^N and Δ^13^C values of Protura were similar to those of some predators (Staphylinidae, Chilopoda, Carabidae).

## Discussion

All three methods used in our study supported the assumption that Protura in their natural habitat actively feed on ECM. In the beech rhizosphere *A. gallicum* incorporated ^13^C but not ^15^N from labelled plants indicating that the species fed on ECM hyphae since plant C, but little plant N, is transferred into mycorrhizal fungi. In fact, ectomycorrhizal root tips were highly enriched in ^13^C and less in ^15^N, whereas lateral roots were less enriched in ^13^C but more in ^15^N, which is in line with the functioning of ECM in capturing soil N and transferring it to plant roots [22]. Our results suggest that C and N in *A. gallicum* originated from different sources. While C is derived from freshly assimilated plant C transported from leaves into roots and into ECM, N is derived from soil via inorganic and organic N compounds derived from decomposing soil organic matter assimilated by ECM hyphae and transported to plant roots [23]. This contrasts other soil invertebrates relying on root-derived resources which use both root C and N for tissue formation [24].

Due to low sample numbers in ash caution is needed in interpreting the findings, however, as compared to beech, *A. gallicum* incorporated markedly less root-derived C in the ash rhizosphere, indicating that *A. gallicum* feeds little on arbuscular mycorrhizal fungi associated with ash. Presumably, Protura switch diet and feed on saprotrophic fungi if ECM are scarce as suggested earlier [13]. However, as indicated by low abundance of *A. gallicum* in the ash as compared to the beech rhizosphere, they suffered from food shortage resulting in low density. Little feeding on arbuscular mycorrhizal fungi by *A. gallicum* likely is related to the smaller hyphal diameter of arbuscular mycorrhizal fungi as compared to ECM [22], suggesting that small size prevents effective consumption of the hyphal cytoplasm. Compared to saprotrophic fungi, arbuscular mycorrhizal fungi are less preferred by fungal feeding soil invertebrates [25, 26], but see [27]. Potentially, arbuscular mycorrhizal fungi are protected against predation by metabolites provided by the plant [28].

The results support earlier undirect evidences that in temperate forests Protura rely on root-derived resources [10, 29] and are specialized in feeding on mycorrhiza [13, 14]. The main predators of Protura are assumed to be Gamasida [30], which is supported by a strong decrease in abundance with increasing numbers of Gamasida [31]. Thus, freshly-fixed root-derived C may be incorporated into small-sized soil predators via an ECM—Protura energy channel but this needs further investigation in controlled experiments.

NLFA analysis added to the results of the stable isotope labelling data suggesting that Protura ingest food resources by sucking on plant based resources. High incorporation of the plant marker fatty acids 16:0 and 18:1ω9 in *A. gallicum* also has been reported for other fungal feeding animals such as the Collembola species *Protaphorura fimata* Gisin, 1952 feeding on the fungus *Agrocybe gibberosa* (Fries) Fayod, 1889 [32]. High concentration of the plant marker fatty acids 16:0 und 18:1ω9 in *A. gallicum* might reflect that via feeding on ECM they incorporate plant fatty acids transported into ECM [33, 34]. In contrast to our hypothesis, *A. gallicum* contained little 18:2ω6,9, a dominant component of the lipid membrane of fungi commonly used as fungal biomarker [35] and reaching high concentrations in fungivorous microarthropods such as *Lepidocyrtus cyaneus* Tullberg, 1871 (21.86 ± 1.64%; S. Bluhm, unpubl. data). However, it has been found that the concentration of 18:2ω6,9 is ten times lower in the NLFA as compared to the PLFA fraction in ECM fungi [34]. This supports earlier observations that, in contrast to Collembola grazing on fungi, *A. gallicum* sucks up the cytoplasm of mycorrhizal hyphae, thereby not ingesting membranes. Furthermore, it has been shown that ECM have distinct fatty acid profiles varying between species [36]. The high concentrations of the bacterial-marker fatty acids 16:1ω7 and 18:1ω7 in *A. gallicum* also may be explained by feeding on ECM, as these biomarkers may also be present in basidiomycetes and arbuscular mycorrhizal fungi [37, 38].

The trophic niche of Protura, as reflected by natural abundances of ^13^C and ^15^N, largely overlapped with that of ECM again supporting the conclusion that Protura feed on ECM. The ^15^N natural abundance in *A. gallicum* in microcosms with unlabelled control plants exceeded the ^15^N natural abundance in ECM root tips by 3.5‰, which is in line with the mean trophic level fractionation of 3.4‰ [39].

Natural variations in stable isotope ratios of Protura differed markedly from those of other soil invertebrates, suggesting that selective feeding on ECM by Protura is unique. Other soil animal taxa such as Onychiuridae (Collembola) also might feed on ECM, but in addition feed on other resources such as root hairs, i.e. are not specialized in feeding on ECM [9, 40]. The fact that the density of Protura typically is low as compared to other mesofauna groups, contrasts the high biomass of ECM in temperate forest ecosystems. This striking pattern indicates that ECM are well protected from grazing by soil invertebrates including Protura, presumably via toxic compounds and crystalline ‘spines’ on the surface of hyphae [28, 41]. High toxin concentrations and exclusive feeding on ECM by Protura likely contribute to their low abundance.

## Conclusions

All three methods used indicate that Protura predominantly feed on ECM by sucking up the cytoplasm of hyphal cells. Specialized feeding on a narrow spectrum of prey contrasts the dominance of generalist feeders in soil animal communities [42, 43]. This also applies to the most abundant soil mesofauna taxa, i.e. Collembola and Oribatida [44]. Limited consumption of ECM hyphae by soil invertebrates might have contributed to the evolutionary success of the plant—mycorrhiza symbiosis and to major ecosystem functions such as plant growth and storage of organic matter in boreal and temperate forest soils.

## Methods

### Field sampling

Samples were taken from study sites in Hainich-Dün, Schorfheide-Chorin and Schwabian Alb, which form part of the “Biodiversity Exploratories”, a large integrative biodiversity project across Germany [45]. To analyse natural variations in stable isotope ratios in Protura, soil cores of a diameter of 21 cm were taken in 2011 from Schorfheide-Chorin and Schwabian Alb, (for details on study sites and sampling see [46]. Animals were extracted using heat [47], collected in diethylene glycol–water solution (1:1) and stored in 70% ethanol until further processing. Selected specimens were mounted on slides in Marc Andre medium and were observed and identified with a phase-contrast microscope based on recent keys [48, 49]. Only samples with ≥ 10 individuals of Protura were used for stable isotope analysis resulting in 16 samples from 9 localities (3 coniferous and 6 beech forests; Additional file 1: Table S1). To analyse NLFA composition of Protura, additional specimens of *A. gallicum* were sampled from an old-growth beech forest near Silberhausen in Hainich-Dün, central Germany, in November 2017. Even though the study site and year of sampling differed from the other experiments the results can be compared and integrated as the trophic position of soil invertebrates is rather constant between different forests and years [50, 51]. Animals were extracted from soil by heat [47]. In total, four samples were taken each containing 30–60 individuals of *A. gallicum*. All individuals were stored in methanol at − 80 °C right after identification.

### Labelling experiment

For analysing the importance of root-derived C and N for *A. gallicum,* samples from a pulse labelling experiment conducted in 2012 were used (for details see [40]). In May 2012 beech (*Fagus sylvatica* L.) and ash (*Fraxinus excelsior* L.) seedlings were excavated with intact surrounding soil and litter from a beech forest near the city of Göttinger (Reyershausen, Germany). The seedlings were transferred into a plant-growth chamber and pulse-labelled with ^13^CO_2_. Further, leaves were immersed into a ^15^N ammonium chloride solution for 3 days (for details see [40]). After 5 and 20 days, animals from litter and soil were extracted by heat [47]. Animals were identified under a dissecting microscope and preserved in 70% ethanol. A subset of five labelled and six control beech seedlings, and two labelled ash seedlings was used for stable isotope analysis (Additional file 1: Table S1). To analyse the transfer of C and N from roots into mycorrhiza, fresh beech fine roots of a diameter < 1 mm were sampled. Soil particles were carefully removed from the roots, mycorrhizal root tips were cut from the root. The root piece before the mycorrhizal root tip were used as second-order lateral root (lateral root), for details see [52]. Then, samples were freeze-dried, ground in a ball mill (Retsch Schwingmuehle MM400, Retsch GmbH, Haan, Germany) and stored in a desiccator until stable isotope analysis.

### Neutral lipid fatty acid analysis

Lipids from freshly-frozen Protura were extracted as described in [32]. In short, neutral lipid fatty acids (NLFA) were dried in a rotation vacuum concentrator, saponified, methylated and washed following the procedures given for the Sherlock Microbial Identification System (MIDI Inc., Newark, NJ, USA; see [35]). Then, the lipid fraction was transferred into test tubes and stored at − 20 °C until analysis via gas chromatography. The gas chromatograph (CLARUS 500, Perkin Elmer, Waltham, USA) was equipped with a flame ionisation detector and an Elite-5 capillary column (30 m × 0.25 mm i.d., 0.25 µm film thickness; Perkin Elmer, Waltham, USA). Fatty acid methyl esters (FAMEs) were identified by comparing retention times of samples with standard mixtures composed of 37 different FAMEs ranging from C11 to C24 and bacterial FAMEs (for details see [53]). NLFAs are given in percentages of total fatty acids extracted from the respective sample. NLFAs 16:1ω7, 18:1ω7 and cy19:0 served as bacterial markers, 18:2ω6,9 as fungal and 18:1ω9 as plant marker [21].

### Stable isotope analysis

For ^13^C and ^15^N measurements of Protura from the labelling experiment and field sites, an appropriate number of animals to reach a measurable minimum of 5 µg dry weight (2–20 individuals; Additional file 1: Table S1) were placed into tin capsules and dried at 40 °C for 48 h. For the labelling experiment six samples of *A. gallicum* for control and five samples for labelled beech and two samples for labelled ash trees were used. For comparing natural abundance data of Protura with those of other soil invertebrates four samples of *A. gallicum*, eleven samples of *Eosentomon* spp. (*Eosentomon armatum* Stach, 1926 and *Eosentomon silvaticum* Szeptycki, 1986) and one sample of *Acerentulus* sp. were used. Stable isotope ratios of animals were analysed using a coupled system consisting of an elemental analyser NA 1100 (CE-Instruments, Rodano, Milan, Italy) and a mass spectrometer (Delta plus, Finnigan MAT, Bremen, Germany) coupled by a ConFlo III interface (Thermo Electron Corporation, Bremen, Germany) [54].

For stable isotope analysis of mycorrhizal root tips and lateral roots from the labelling experiment 1–2 mg dry weight were filled into tin capsules and analysed as described above but using another elemental analyser (NA 1108; Fisons-Instruments, Rodano, Milan, Italy). Abundances of ^13^C and ^15^N are expressed using the δ notation with δ_sample_ [‰] = [(R_sample_ − R_standard_)/R_standard_] × 1000, with R_sample_ and R_standard_ representing the ^13^C/^12^C and ^15^N/^14^N ratios of samples and standard, respectively. For ^13^C Vienna PD Belemnite (V-PBD) and for ^15^N atmospheric nitrogen served as the primary standard. Acetanilide (C_8_H_9_NO, Merck) was used for internal calibration. In the labelling experiment, enrichment of ^13^C and ^15^N in Protura, mycorrhizal root tips and lateral roots of labelled treatments were expressed as Δε‰ ^13^C = δ^13^C _label_ − δ^13^C_control_, and Δε‰ ^15^N = δ^15^N_label_ − δ^15^N_control_. Natural abundances of C and N stable isotopes in Protura are expressed as litter-normalized Δ^13^C and Δ^15^N values by subtracting δ values of the mixed litter samples from δ values of animals to account for inter-site variability [55].

### Statistical analysis

Statistical analyses were performed using R v. 3.4.3 (R Core Team 2017). In the labelling experiment C and N enrichment of *A. gallicum* were analysed separately using single-factor analysis of variance (ANOVA) to inspect differences in Δε^13^C and Δε^15^N values between samples from labelled and control tree seedling species. To inspect differences between the two compartments lateral root and mycorrhizal root tip Δε^15^N and Δε^13^C values were analysed separately using linear mixed effects models. A random effect of root tip identity (root ID) avoiding pseudo-replication of compartments of the same root tip was included. To increase homogeneity of variances Δε values of Protura and roots were log-transformed.

Litter-normalized Δ^13^C and Δ^15^N values of Protura species were inspected using Welch Two Sample t-test. In addition, litter-normalized Δ^13^C and Δ^15^N values of Protura were compared with published data on mycorrhizal and saprotrophic fungal sporocarps from coniferous forests [56] and other soil invertebrates from deciduous forests [46]. The dataset from Trudell et al. [56] included 105 ECM and 44 saprotrophic species. The dataset from Klarner et al. [46] included 125 species of soil invertebrates from dominating taxonomic groups including Oribatida, Mesostigmata, Araneae, Collembola, Lumbricidae, Diplopoda, Chilopoda and others.

## Additional file

**Additional file 1.** Experimental design. Location, habitat, sample number and details for analysed Protura of the three methods (labelling experiment, neutral lipids and natural stable isotopes).
